# Case Report: Implementation of a Multi-Component Behavioral Health Integration Program in Obstetrics for Perinatal Behavioral Health

**DOI:** 10.3389/fpsyt.2021.734883

**Published:** 2021-11-23

**Authors:** Heather Flynn, Megan Deichen Hansen, Amandla Shabaka-Haynes, Shay Chapman, Kay Roussos Ross

**Affiliations:** ^1^Department of Behavioral Sciences & Social Medicine, College of Medicine, Florida State University, Tallahassee, FL, United States; ^2^Florida Department of Health, Tallahassee, FL, United States; ^3^Department of Obstetrics & Gynecology, College of Medicine, University of Florida, Gainesville, FL, United States

**Keywords:** perinatal, behavioral health, obstetrics, psychiatric consultation, mood anxiety

## Abstract

Despite growing research and policy attention, perinatal behavioral health conditions (i.e., mental health and substance use disorders) remain prevalent, burdensome for families, and largely untreated in the US. Researchers have documented an array of barriers to accurate detection, linkage with effective treatment, and improved outcomes for perinatal women with behavioral health disorders. It is clear that a multi-component approach that integrates evidence-based detection and management of perinatal behavioral health in the context of obstetrics care can be effective. This paper presents the initial development of a clinical quality improvement program that includes evidence-based components of behavioral health integration in obstetrics in the state of Florida in the US. The FL BH Impact (Improving Maternal and Pediatric Access, Care and Treatment for Behavioral Health) program, guided by the RE-AIM model for program implementation, has been developed over the past 2 years. Program components, initial implementation, and preliminary findings are presented. Following the implementation phase, the program has enrolled 12 obstetrics practices and 122 obstetrics providers in program engagement and training activities. The primary program component allows for obstetrics clinician telephone access to a statewide listing of behavioral health referral resources for patients and access to consultation with psychiatry. Since program implementation, the program has received a total of 122 calls to this line, with an expected increasing trajectory of calls over time. Results suggest this program is feasible to implement across a large geographic area. Challenges to implementation and future directions are discussed. These types of multi-component approaches to improved management and outcomes for perinatal behavioral health are promising and must be expanded and sustained in the US.

## Introduction: Description of the Problem

Perinatal behavioral health conditions (i.e., mental health and substance use disorders) occurring during pregnancy and the time following birth are serious but treatable mental health conditions that affect a large proportion of US women. Approximately 10–20% of women will experience perinatal depression (1). Additionally, while rates of perinatal anxiety disorders have been reported variably in the literature, comprehensive reviews suggest that they occur among approximately 8–20% of women (2–4), and, along with substance use and misuse, often occur concomitantly with depression. Anxiety symptom measures and longer assessment periods reveal higher rates than diagnostic measures and shorter assessment intervals. Despite these prevalence rates, most postpartum women do not receive professional help (5, 6). Reasons for the relatively low rates of adequate perinatal behavioral healthcare are multifaceted and occur at multiple points of care. Barriers to care, for example, can include system- and provider-related issues, such as lack of training for obstetricians and other prenatal care providers, screening, treatment, and monitoring of perinatal behavioral health conditions, limited behavioral health referral networks, and temporal barriers, such as short visit times and, especially during the postpartum period, limited patient contact (7). Other barriers to care stem from patient-related issues, such as perceived stigma related to perinatal behavioral health conditions, concerns related to safety and efficacy of psychiatric care during pregnancy and breastfeeding, and difficulty navigating their multiple care needs during an already vulnerable life stage (7). Practical barriers include concerns related to affordability of care, time restrictions, lack of transportation, and lack of childcare (7, 8), and as such, contribute to disparities among women who are able to access behavioral health treatment and those who are not.

This gap in care can lead to dangerous implications for new mothers and their families. When women are unable to access necessary behavioral health care, new or worsening mental health conditions may go un-/under-detected, and women may go without access to evidence-based treatment. Undetected and untreated behavioral health conditions can have serious and lifelong ramifications for women, including the experience of chronic mental health and substance use conditions and increased incidence of comorbid physical health conditions (9). Children and family members of women with behavioral health conditions may also experience a range of social, emotional, psychological, and developmental disadvantages (10–14).

Given the existing gap in perinatal behavioral healthcare provision and the serious adverse effects of untreated maternal behavioral health conditions, there is a need to expand capacity for identifying and addressing women's behavioral health needs at the point of prenatal care. Critically, the multitude of barriers to care require a multi-systemic approach to perinatal behavioral health care in order to address women's needs in an efficient and effective manner. Integration of detection and management of perinatal behavioral health issues within prenatal care settings is emerging as an effective approach to reducing barriers to care. For example, the Massachusetts Child Psychiatry Access Project for Moms (MCPAP for Moms) is a model for care integration that combines (1) resource and referral support with (2) psychiatric consultation for obstetrics clinicians (physicians, midwives, and nurse practitioners), and (3) provider training regarding the screening, treatment, and ongoing management of maternal behavioral health conditions. Through their program, MCPAP for Moms has trained approximately 70% of Massachusetts' obstetric care providers in best practices for screening, treating, and managing maternal mental health and substance use conditions (15). Following MCPAP for Moms' success in Massachusetts, the Health Resources and Services Administration (HRSA) funded seven states, including the state of Florida, to implement similar perinatal care integration models. The purpose of this article is to present a community case study on the development of a program in Florida designed to enhance perinatal behavioral health care in obstetrics practices. This community case study provides a description of the development and implementation of the Florida BH Impact (Improving Maternal and Pediatric Access, Care and Treatment for Behavioral Health) program, along with the preliminary process and outcomes measures that point to the program's early success. The primary aim of this paper is to document the feasibility of standing up (i.e., developing the infrastructure and implementing key components) the clinical quality improvement program in the northern part of the state and present preliminary findings on the program implementation activities. Initial program successes, challenges, and next steps are highlighted.

### Case Context (Setting and Population)

The state of Florida is the third largest and one of the most diverse states in the US. Annually, the state reports approximately 220,000 births. The sprawling demography and diverse population have presented numerous challenges to perinatal healthcare provision, particularly with respect to women with complex perinatal needs, such as those with behavioral health conditions. These challenges are further compounded by the healthcare policies and overarching system issues, which can make care difficult for women to access. For example, Florida has not adopted Medicaid Expansion. Nearly half (47%) of Florida's births in 2019 were covered by Medicaid, and until 2021, Medicaid benefits expired after 60 days postpartum. Loss of health care coverage for postpartum women in Florida has substantially limited access to health care. Florida recently ranked 49^th^ out of the 50 US states related to behavioral healthcare access for women and 44^th^ for adequate prenatal care provision (16, 17).

These contextual factors significantly contribute to the insufficient behavioral health care of Florida's perinatal population. Women who experience comorbid behavioral health conditions or who experience more stigmatized behavioral health conditions in and of themselves are likely to encounter even greater difficulty seeking and accessing the necessary care. Given the significant negative outcomes associated with undertreated perinatal behavioral health conditions, these findings necessitate enhanced behavioral health service provision on a large scale.

## Framework for Implementing Florida's Enhanced Behavioral Healthcare

In response to the state's need for improved integration of behavioral health in prenatal care settings, the Florida BH Impact Program implemented a multi-prong strategy for enhancing perinatal behavioral healthcare as a clinical quality improvement project beginning in 2019. Using the MCPAP for Moms model as a guide, BH Impact developed and implemented a perinatal behavioral health integration program that aligned with the needs of Florida's large and diverse population of new mothers. FL BH Impact began implementation efforts in a stepwise fashion, first implementing the program within three regions of Northeast Florida (11 of 67 counties), and gradually expanding implementation to other regions of the state, based on provider demand. Implementation within Northeast Florida is significant; this area of the state is characterized by pervasive race-based perinatal health disparities and is home to a large portion of rural residents who experience greater degrees of difficulty accessing necessary perinatal care.

Implementation of the clinical quality improvement program is guided by the RE-AIM model (Reach Effectiveness Adoption Implementation Maintenance). RE-AIM has been developed and used as an effective framework to guide the successful implementation of interventions and programs in public and community programs and sectors since 1999 (18). The components of RE-AIM include: Reach (the number and representativeness of key individuals who are willing to engage in the program); Effectiveness (the impact of the interventions and program on key outcomes); Adoption (the effectiveness of the appropriate number and kind of individuals who initiate the program), Implementation (the intervention agent's fidelity to the key elements of the program), and Maintenance (the organizational sustainability of the program over time). To date, the RE-AIM model has served as a guide for all aspects of our implementation of the BH Impact program, from determining the appropriate stakeholders to engage to identifying process and outcome measures that align with the overarching RE-AIM framework (i.e., measures that evaluate the consistency of intervention implementation and that target intervention efficacy).

Using the RE-AIM framework, the BH Impact project applied a pragmatic approach to intervention implementation, which includes a collaborative partnership between clinicians, researchers, and patient advocates. BH Impact is staffed by a combination of clinical providers and research staff. In addition to BH Impact staff, programmatic content is developed in an iterative fashion, which is informed by clinical providers working in perinatal and behavioral health clinics at each stage of the implementation process.

Implementation efforts were driven by the program's aspirational vision, which is that “no perinatal woman in the project's targeted regions will be untreated for perinatal behavioral health disorders.” In order to address the program's vision, initial implementation efforts required a multi-systemic approach to addressing area-specific barriers and facilitators to care, including (1) training and resource provision for front line perinatal care providers; (2) access to psychiatric consultation and referral resources for perinatal care providers; (3) training for obstetric and behavioral health clinicians in best practice treatments for perinatal behavioral health disorders; (4) support for universal screening for all pregnant and postpartum women using valid behavioral health screening tools (i.e., depression, anxiety, and substance use screening tools) Each of these approaches were offered at no cost to participating behavioral health/obstetrics providers.

## Program Structure

Project leadership included three core members; a program director; a resource and referral specialist; and a psychiatrist consultant who is triple-boarded in gynecology/obstetrics, psychiatry, and addiction medicine. Project activities were further supported by research associates. Project staff are all supported by the grant and aided in project implementation, including data collection and tracking, updating of area-specific referral sources, development of outreach materials, and ongoing communication with engaged practices. Additionally, in an effort to customize the MCPAP for Moms model to the state of Florida's perinatal behavioral health integration needs, BH Impact collaborated with several stakeholders in the implementation region. Stakeholders included the Florida Maternal Mental Health Collaborative (FLMMHC), Florida Association of Healthy Start Coalitions, Center for Health Equity, and the Florida Department of Health. Massachusetts Child Psychiatry Access Project for Moms leadership served as consultants throughout the project. Meetings occur with all of these stakeholders on a regular basis (e.g., biweekly) throughout the year, including bi-weekly meetings with MCPAP for Moms. This programmatic structure provided supports necessary for implementing each of the core project elements and for iterative shaping of the program. Each of the following components has been customized to the needs of Florida and specific enrolled practices based on input from Florida prenatal care providers and perinatal behavioral health stakeholders among the partner groups listed above in the regular meetings.

## Practice Engagement

The RE-AIM implementation framework was used to engage obstetric practices across three main North Florida regions (Gainesville, Jacksonville, and Tallahassee, and surrounding areas), providing coverage for 11 total counties. Prenatal care clinics and providers, not patients, are considered participants in this program. Obstetrics practice outreach activities included multiple strategies, including (1) dropping off or mailing program information folders, (2) sending an e-newsletter monthly or bi-weekly, (3) presentations at statewide or regional meetings/conferences attended by obstetricians, and (4) telephone and in-person meetings with key clinic staff to provide information and answer any questions about the program. Engagement with practices was accomplished in a stepwise fashion, based on overarching practice interest in the BH Impact services, starting with less intensive approaches (e.g., mailing materials) and moving to more intensive approaches based on response (e.g., stopping by the practice).

Initial practice engagement included presentations at ACOG district XII conferences, hospital grand rounds, postal distribution of psychoeducational and program promotion materials, circulation of monthly e-newsletters, and outreach to known practices offering initial training for physicians, nurses, and residents. Any practice located in one of the 11 counties that provide prenatal care was eligible to enroll. Upon receipt of outreach materials, practices were enrolled in the BH Impact program by completing an enrollment form and were eligible for ongoing resource and referral support along with telepsychiatric consultation services. Data collected at enrollment included: practice contact information, name and contact information of the medical or clinical director, the approximate number of deliveries annually, and the number of obstetrics providers (obstetrician, nurse practitioner, midwife). Following enrollment, the initial program orientation and perinatal depression training session were scheduled. The first training focused on depression due to its prevalence and based on statewide interest in perinatal depression. Subsequent training focused on perinatal anxiety and/or substance use based on the clinic's preferences. All training was conducted by the perinatal psychiatrist, who is also board certified in obstetrics/gynecology and addiction medicine. Between training, ongoing technical assistance was offered intermittently, based on practice needs and desires. For example, technical assistance has been offered in the selection, use, and workflow integration of validated behavioral health screening tools. As a result of the COVID 19 pandemic, all clinic engagement, training, and technical assistance contacts shifted from in-person to virtual. In addition, obstetrics providers were reporting significant stress themselves. As a result, the program psychologist and psychiatrist integrated a webinar focused on clinician self-care and mental health. We also began including relevant and up-to-date COVID 19 related information in our biweekly newsletters and our website.

### Resource and Referral Support

Resource and referral support was provided to all enrolled providers/practices. The state of Florida has a uniquely robust open-source online statewide referral database. This referral database was developed by the program staff prior to the onset of this FL BH Impact program in order to assist any point of contact for pregnant and postpartum women in Florida to refer patients to qualified and active behavioral health providers throughout the state. Practices and providers enrolled in the BH Impact program were provided referral and resources via two main mechanisms: (1) live assistance in the use of the statewide behavioral health referral database of perinatal behavioral health providers and (2) real-time telephone access to a referral specialist. The maternal behavioral health referral database includes online access to a geocoded aggregate listing of active and qualified maternal mental health and social service providers (*n* = 1,253) and allows for providers to be searched based on insurance type, geographic proximity to the patient, and provider specialization, among other details. This database is maintained and refreshed on an ongoing basis. In addition to the database, BH Impact enrolled obstetrical providers were given a toll-free telephone number which they could call during normal business hours for real-time provision of area-specific resources for mothers at-risk of behavioral health conditions.

### Telepsychiatric Consultation

Using the toll-free telephone line, obstetrics providers can also access a reproductive psychiatrist. This consultation program is a provider-to-provider consultation, available to all enrolled providers and is conducted throughout regular business hours. The consultation program allows enrolled providers the opportunity to seek evidence-based consultation on diagnoses, treatment, and management of perinatal behavioral health issues, including the safety and effectiveness of pharmacotherapy options for pregnant and lactating women. By providing consultative services, BH Impact aims to empower obstetrical clinicians in their ability to expand and enhance their capacity to address perinatal behavioral health disorders.

## Program Adoption and Initial Findings

All program information is recorded and tracked using a secure clinical data management system. All program outcomes are directly related to the uptake of program components listed above. Patient outcomes are not tracked as part of this program since the primary focus is on educating and equipping obstetrics providers to address perinatal behavioral health in their practices. Therefore, tracked data are included in the Tables and include all information related to training and technical assistance contacts with clinical sites, number and nature of calls to the program, referral resources provided to the calling clinician, and the type of clinician using the service (e.g., obstetrician, nurse practitioner). During the first 2 years of the BH Impact Project's pilot implementation, a total of 12 healthcare practices have been enrolled, including eight obstetric practices (Table 1; RE-AIM Reach). Among enrolled practices, 123 providers and 350 individuals (including clinic support staff) have been trained (Table 2). As can be seen in Table 2, a total of 18 training activities have been conducted in just under 2 years of the program development (RE-AIM Implementation). Training included information on diagnosis, prevalence screening, treatment, and management of perinatal depression, anxiety, and/or substance use delivered by our psychiatrist consultant. In addition, the trainings were used as an opportunity to orient trainees about the offerings of the FL BH Impact Program and emphasized the availability of the psychiatric consultation/resource and referral line available to providers during normal business hours. Despite the fact that the initial stage of the program was mainly aimed at building the program infrastructure, identification of obstetrics practices to target and outreach, and enrollment and training of those practices, we received 122 provider consultation and referral/resource calls (RE-AIM Adoption) in under 2 years (approximately 6.4 calls per month).

**Table 1:** Clinical practice enrollment by type, location, and number of providers enrolled between October 1, 2019 to June 30, 2021.

**Type**	**Total number of enrolled practices**	**Region**
Family practice	1	Leon
Inpatient obstetrics	2	Duval, Leon
Outpatient obstetrics	8	Alachua, Duval, Leon, Orange, West Palm
Outpatient Psychiatry	1	Alachua
Total	12	
**Total number of clinicians across all practices**	**133**	

**Table 2:** Provider training activities by type, number of activities and number of providers engaged between October 1, 2019 to June 30, 2021.

**Type**	**Total number of activities**	**Total number of providers trained**	**Total people (including support staff) trained**
Practice level training	13	92	230
Grand rounds training	3	29	45
Technical assistance engagement	2	2	75
Total	18	123	350

**Figure 1:**
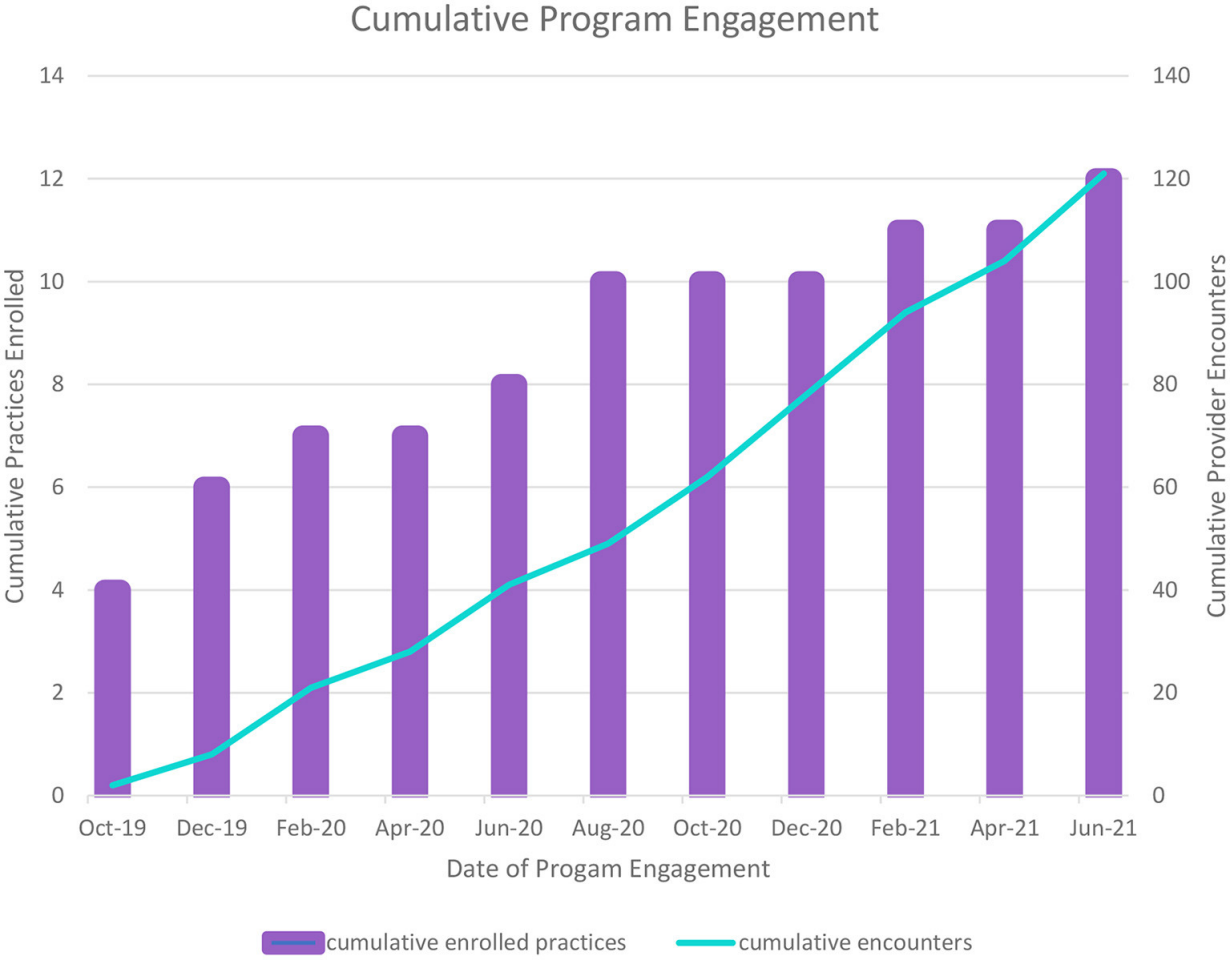
Implementation of a multi-component behavioral health integration program in obstetrics for perinatal behavioral health.

As can be seen in Table 3, a mix of obstetrics provider types used the consultation and referral line (RE-AIM Adoption). The types of patient issues that were of concern for the calls were also diverse, as can be seen in Table 4, with the majority being comorbid perinatal depression plus anxiety. Among the 9% of calls pertaining to substance use, the majority of those (73%) were noted to have co-occurring depression, anxiety, or both. The Figure 1 shows the trajectory of calls to the psychiatric consultation/resource and referral line over time. As expected, the trajectory of the number of calls to the line showed an upward increase over time as ongoing dissemination of information about the program and additional training and technical assistance activities were provided to the providers.

**Table 3:** Encounters according to provider type and encounter type from September 26, 2019 to June 30, 2021.

**Provider type**	**Total number of encounters^a^**	**Telehealth clinician support**	**In-person clinician support**	**Percent of total (%)**
Obstetrician	26	19	7	21.3
Trainee (fellow or resident)	17	11	6	13.9
Midwife	24	21	3	19.7
Physician assistants/Nurse practitioner	40	25	15	32.8
Other	15	12	3	12.3
Total	122	88	34	

a *Includes hallway encounters, email and follow-up consultations*.

**Table 4:** Encounters according to reported behavioral health condition from September 26, 2019 to June 30, 2021.

**Conditions**	**Total number of encounters**	**Percent of total (%)**
Perinatal mood disorder	27	22.1
Anxiety and related disorders	14	11.5
Co-occurring perinatal mood and anxiety disorders	35	28.7
Psychotic disorder	4	3.3
Substance use disorders^*^	11	9
Other i.e., community referral resource, follow up consults	16	13.1
Birth trauma	2	1.6
Unknown	13	10.7
**Total**	**122**	

* *72.7% of patients diagnosed with substance use disorders were also diagnosed with perinatal mood disorders (9.1%), anxiety and related disorders (18.2%), or both (45.5%)*.

In addition to documentation of the program components uptake (i.e., practice enrollment, training, use of consultation/resource, and referral line), active efforts to collect information on the use of validated screening tools for depression, anxiety, and substance use among enrolled practices are underway. Although BH Impact provides training and technical assistance on the use of validated screening tools, each practice acts autonomously, and individual screening processes vary from practice to practice. The program has worked to identify a point person at each site who can assist with providing the number of patients who have been screened using a validated tool during a specified time period. Although this is one of the more challenging aspects of the program, we have successfully obtained screening numbers from two practices to date. Based on data collected to date, a total of 1,178 women have been screened across the two practices with validated tools depression screening tools (RE-AIM Effectiveness). Women at these practices are screened universally during the postpartum period, and prenatal screening time frames vary. Ongoing efforts to encourage the use of validated tools to screen for anxiety and substance use are underway.

## Discussion

This community case study report describes the initial implementation of a behavioral health integration program for obstetrics providers. The major components of the program include enrollment of obstetrics practices and providers within our catchment area, obstetrics provider training and technical assistance in behavioral health, access to psychiatric consultation and referral resources for obstetrics providers, and universal behavioral health screening support. The Florida BH Impact Program shares major components with the MCPAP for Moms program, which is a population-based perinatal psychiatric program in Massachusetts that includes training and toolkits for perinatal providers and staff, telephone access to psychiatric consultation and care coordination, including linkages for patients with behavioral health referrals and resources. MCPAP for Moms has been shown to have strong feasibility and uptake statewide, improved EPDS scores, and sustainable cost outcomes (15, 19). Several other states have begun to implement similar behavioral health integration programs in obstetrics. Although FL BH Impact shares several components with MCPAP for Moms (access to psychiatric consultation, obstetric provider training and technical assistance, and referral resources for obstetrics providers), this program has some key differences adapted to the needs of Florida. First, our program grew out of 3 years (2015–2018) of information gathering through the FLMMHC, which is a statewide coalition of perinatal mental health stakeholders including clinicians, researchers, advocates, and agency representatives that met monthly between 2015 and 2018 to identify gaps and needs for this population. Second, given the size and diversity of the state, the FL BH Impact program targeted the northern region of Florida and is not offered statewide. Third, since many of our targeted regions are HRSA designated psychiatry shortage areas, our program has focused on the training of behavioral health providers in addition to obstetrics providers from the outset. Fourth, since the behavioral health system in Florida is very fragmented and addressed by multiple state agencies, our behavioral health resource and referral directory is available to the public in order to facilitate open access. In Massachusetts, the resource and referral directory is only available to enrolled practices. Fifth, given the racial and ethnic diversity in the state, our program was developed with a multi-pronged plan to address perinatal health disparities.

Guided by the RE-AIM model, this paper describes the program implementation and initial results of the Florida Program. Programs that include multiple components that align with documented perinatal behavioral health care needs at various care junctures are critical based on the low rates of adequate behavioral health treatment among women in prenatal care contexts. For example, several studies have shown that behavioral health screening and referral as recommended by ACOG (20–22) and other bodies have shown no impact on depression outcomes (5, 23) in the absence of additional supports or components. Based on a recent review of evidence-based approaches to improving perinatal depression care and outcomes, a comprehensive, multi-component approach is needed (24). Components of a care pathway likely to be most effective in improving outcomes include (1) screening, (2) assessment, (3) triage and referral, (4) access to treatment, (5) treatment initiation, (6) symptom monitoring, and (7) treatment adjustments based on response (22). The FL BH Impact program has implemented the first five components of this recommended care pathway, and future work will build on these components to address the treatment outcome aspects. Full population-based implementation of such a model holds promise to drastically reduce the prevalence and burden of perinatal behavioral health disorders.

Based on the first 2 years of standing up the FL BH Impact program and initial findings, it is clear that enrollment, engagement, training, and obstetric provider use of the program consultation and resources is feasible, as indicated by the program uptake data presented in the Tables. We have fully engaged a total of 12 obstetrics practice and 123 obstetrics providers in our first 2 years of development. Despite the fact that the first several months of the program focused on foundational program infrastructure development (e.g., hiring and training of staff, creation of a database, institutional contracting, and compliance), we have seen an expected increasing trajectory of use of the psychiatric consultation/resource and referral service, with an average of just over six calls per month. Notwithstanding the COVID 19 pandemic occurring in year two of the program, we have successfully conducted 18 different training encounters with the practices so far. Based on the nature of the psychiatric consultation and referral resource calls, it is clear that the majority of obstetrics providers are seeking guidance for women with co-occurring depression and anxiety. Among calls for substance use, the majority of those included patients with co-occurring depression and anxiety. Although further examination is needed, it appears that obstetrics providers are seeking assistance in managing more complex perinatal patients (i.e., patients with co-occurring behavioral health conditions). This preliminary finding suggests that integration of psychiatric consultation, access to referral and resources, and screening support may indeed enhance the ability of obstetrics providers to manage perinatal behavioral health disorders as long as adequate supports are in place.

One of the biggest challenges with the implementation of these types of behavioral health integration programs in obstetrics or other primary care settings is the engagement and participation of physicians and other clinicians in the sites. Despite the fact that we have conducted numerous types of clinician outreach efforts, it can be challenging to realize full uptake of the program while clinicians face a multitude of demands on their time. Although perinatal depression screening is recommended by ACOG (20), rates of actual screening in practice are in the 44–65% range (5, 25–27). FL BH Impact has begun to address this by sending biweekly newsletters to the clinicians, reminding them of the services we offer at no cost, as well as scheduling follow-up training and technical assistance contacts with the sites. We will explore the effect of clinic financial incentives in the coming year. Although we were able to obtain the actual numbers of women screening with at least one validated depression tool from two practices that aim to screen all prenatal patients at least once (*n* = 1178), efforts to obtain actual screening numbers and percentages screened among all prenatal care-seeking patients has been challenging. Future directions will focus on patient outcomes such as rates of screening and linkage with treatment. This challenge relates to the overall difficulty keeping ongoing engagement of the obstetrics practice clinicians and staff. As described in a previous report that outlines strategies to implement screening on obstetrics practices, it is clear that ongoing contacts and trusted, mutually beneficial relationships with at least one behavioral health champion at the site does increase engagement (28). Future efforts should also engage pediatric practices, as many women do not see a primary care provider in the postpartum period but will encounter a pediatrician.

Another ongoing effort for the FL BH Impact program involves the administration and collection of provider self-efficacy data. Collection and analysis of self-efficacy around key aspects of detection and management of perinatal behavioral health providers will be important in understanding whether and how these integration programs can impact the ability of obstetrics providers to expand their scope of practice to include patients with recurrent or comorbid perinatal mood and anxiety disorders.

A final and critical challenge for these multi-component care models is sustaining the programs in the absence of grant funds. It is vital that insurance providers such as Medicaid and other private insurance providers recognize the emerging economic impact data, including costs per patient of the programs along with the return on investment. Most states in the US already have reimbursement codes for behavioral health screening, and Massachusetts has payers that reimburse psychiatric consultation components. These reimbursement and payment models must account for all evidence-based components in order to realize the impact on clinical and functioning outcomes for women and families.

The initial implementation phase of FL BH Impact has shown feasibility in building this program in several regions in northern Florida and increasing usage of the program among our enrolled obstetrics providers. This initial phase has taught us that the outreach and partnerships with obstetrics providers are key to success and attempts to continue to address this challenge are ongoing. In addition, the development, updating, and dissemination of a statewide perinatal behavioral health referral and resource directory have been instrumental. Having this resource available has been a clear incentive for obstetrics providers to engage. The next steps of the project will include administration and collection of provider self-efficacy measures, expanding training of behavioral health providers in evidence-based treatment of behavioral health, focus on patient outcomes, and expansion of the program to other regions of Florida. Programs similar to FL BH Impact that include multiple components that address the array of barriers and needs documented in the perinatal behavioral health care literature over the years have the potential to drastically increase the number of affected women who get the care they need and to ultimately reduce the prevalence and burden of perinatal mood and anxiety disorders. Implementation of universal screening, obstetrics clinical access to training resources and psychiatric consultation, sustainment of a behavioral health referral database, and monitoring of treatment outcomes pose challenges, but preliminary results show uptake of the program among enrolled providers. Similar programs are developing throughout the US and must be evaluated based on feasibility, implementation, effectiveness, and sustainability.

## Data Availability Statement

The raw data supporting the conclusions of this article will be made available by the authors, without undue reservation.

## Ethics Statement

The studies involving human participants were reviewed and approved by Florida State University Institutional Review Board. Written informed consent for participation was not required for this study in accordance with the national legislation and the institutional requirements.

## Author Contributions

All authors listed have made a substantial, direct and intellectual contribution to the work, and approved it for publication.

## Funding

This project was funded by the Health Resources and Services Administration (HRSA) of the U.S. Department of Health and Human Services (HHS) under grant number UF5MC26845.

## Conflict of Interest

The authors declare that the research was conducted in the absence of any commercial or financial relationships that could be construed as a potential conflict of interest.

## Publisher's Note

All claims expressed in this article are solely those of the authors and do not necessarily represent those of their affiliated organizations, or those of the publisher, the editors and the reviewers. Any product that may be evaluated in this article, or claim that may be made by its manufacturer, is not guaranteed or endorsed by the publisher.
